# Low-Grade Adenosquamous Carcinoma of the Axilla of Breast Origin in a Male: A Case Report and Literature Review

**DOI:** 10.3389/fonc.2020.01714

**Published:** 2020-10-30

**Authors:** Xingqiang Yan, Fanshuang Zhu, Qiupeng Wang, Lijie Chen, Yixing Zhou, Zenggui Wu, Linhang Mei, Zhaosheng Ma, Binbin Cui, Feilin Cao

**Affiliations:** ^1^Department of Surgical Oncology, Taizhou Hospital, Wenzhou Medical University, Wenzhou, China; ^2^Department of Pathology, Taizhou Hospital, Wenzhou Medical University, Wenzhou, China

**Keywords:** breast, cancer, estrogen receptor, low-grade adenosquamous carcinoma, metaplastic carcinoma

## Abstract

Low-grade adenosquamous carcinoma (LGASC) is a rare invasive tumor that occurs in breast parenchyma. It has previously only been reported in females. Herein, we describe the case of a 52-year-old male who presented with a palpable mass in his right axilla that he reported had been present for 20-years. This is the first report of a male patient with LGASC. Core needle biopsy pathology revealed a benign mass of mammary origin, but its type was initially misdiagnosed. It was only correctly identified via postoperative pathology after local excision, which indicated that the mass exhibited the typical pathological characteristics of LGASC. Immunohistochemical analysis revealed positive expression of estrogen receptor, which was inconsistent with the typical “triple-negative” immunophenotype of LGASC. After resection of the mass the patient was advised to participate in regular outpatient follow-up. In conclusion, LGASC should be considered in male patients with a mass lesion in their breast or axilla, even when core needle biopsy indicates a benign mass of breast origin. One-stage local resection is recommended for the treatment of male patients with LGASC, but it is crucial to ensure that the margins are negative and postoperative adjuvant radiotherapy is not recommended.

## Introduction

Low-grade adenosquamous carcinoma (LGASC) is an extremely rare neoplasm that occurs in breast parenchyma. It is classified as a subtype of metaplastic carcinoma (1). LGASC occurs at any age in females, but it has never been reported in a male (2–4). There are no typical clinical or imaging features of LGASC, it is diagnosed based entirely on its characteristic histopathology (3, 5). In terms of immunohistochemistry, it usually exhibits a so-called “triple-negative” immunophenotype, which refers to an absence of estrogen receptor (ER), progesterone receptor (PR), and human epidermal growth factor receptor 2 (HER2). The progression of LGASC is very slow and its prognosis is excellent, unlike those of other triple-negative breast cancers (1–4). Herein, we describe the case of a male patient with LGASC who presented with a palpable axillary mass. According to the available literature, this is the first report of a male patient with LGASC.

## Case Report

**Figure 1:**
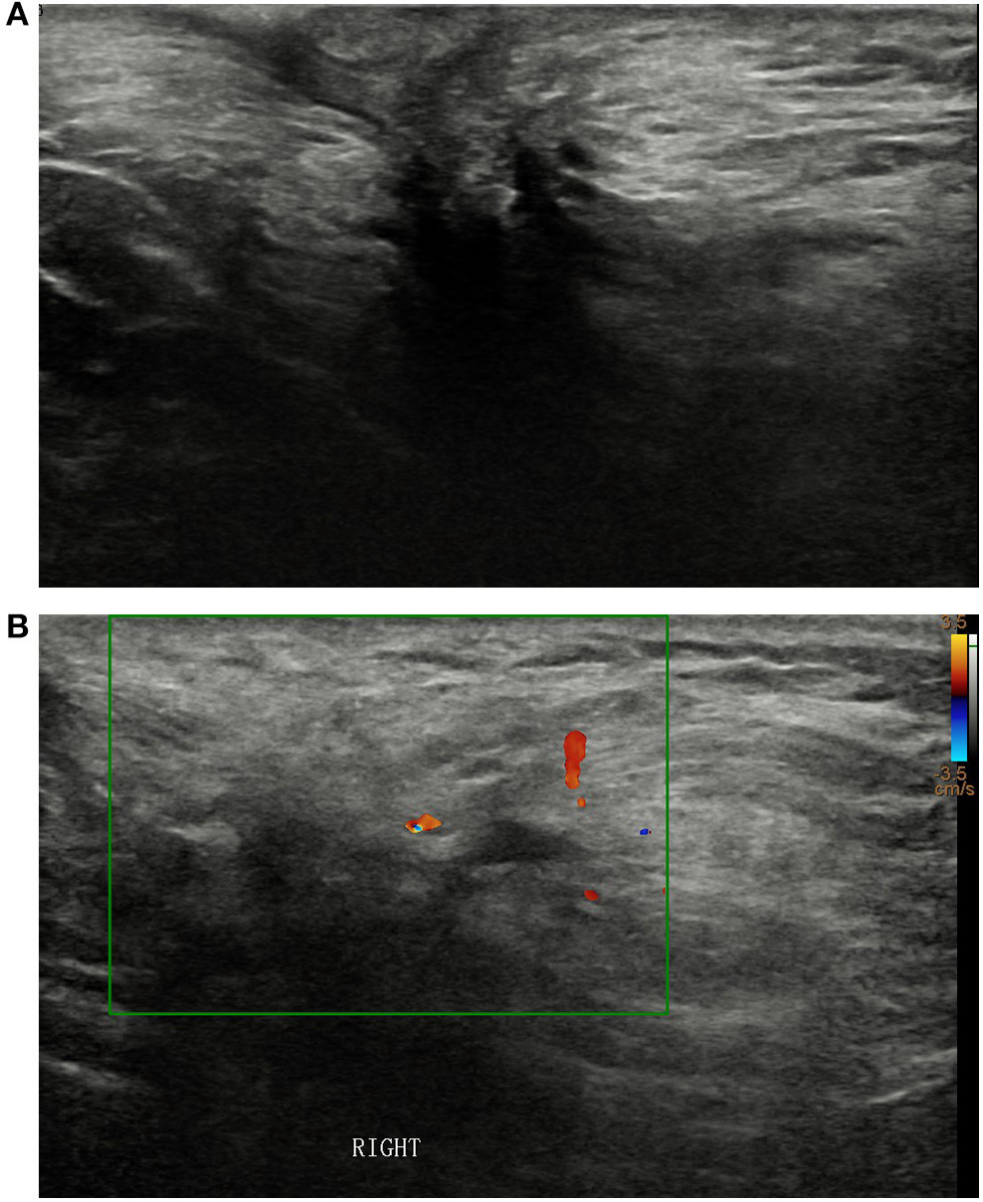
**(A)** Sonographic examination of the right axilla revealed an irregularly shaped hypoechoic mass with unclear borders and uneven internal echoes. **(B)** Color Doppler ultrasonography **(B)** depicted spot-like blood flow signals around the mass.

A 52-year-old male presented with a palpable mass in his right axilla that he reported had been present for 20-years. Sonographic examination revealed an irregularly shaped hypoechoic mass with unclear borders, uneven internal echoes, and dimensions of ~3.4 × 3.4 × 1.9 cm in the right axilla (Figure 1A). Color Doppler ultrasonography depicted spot-like blood flow signals around the mass (Figure 1B). The imaging examinations did not depict any signs of breast tissue in either of the axillae or the chest. The patient had no breast cancer risk factors such as gynecomastia and no apparent family history of breast cancer. Physical examination revealed a mass with a hard texture, unclear borders, poor mobility, no tenderness, skin concavity, and dimensions of ~4.0 × 3.0 cm in the right axilla. No abnormalities were detected in the left axillary area or in the breast parenchyma on either side.

**Figure 2:**
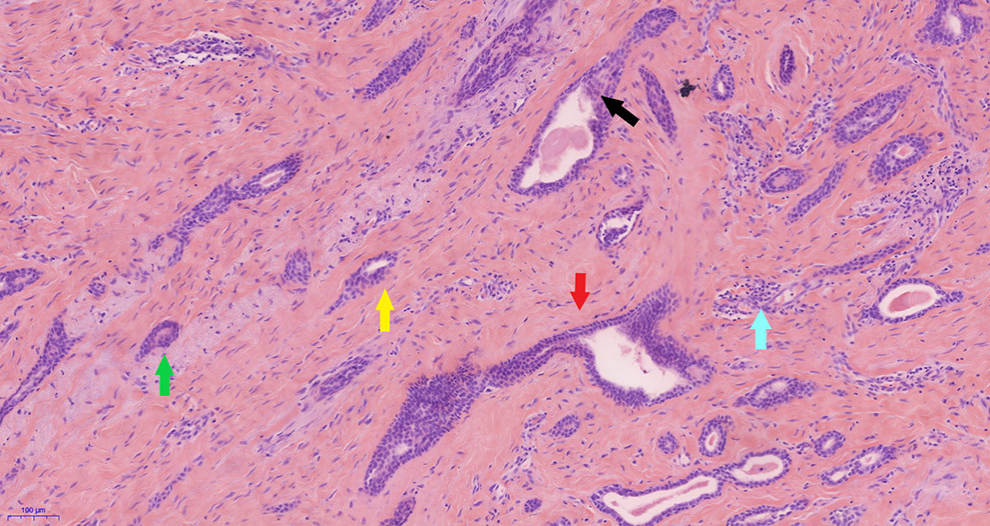
Pathology of the low-grade adenosquamous carcinoma. In photomicrography (original magnification ×100) scattered small glandular ducts and nests of squamous differentiated cells were evident in the sclerosing stroma. The glands were elongated, with angulated (red arrow), comma shaped (green arrow), or polliwog-shaped (yellow arrow) appearances in a disordered infiltrative pattern. The nests of squamous cells (black arrow) were mostly solid bands, and some of them formed keratocysts, of various sizes. Mitosis was rare. Peripheral lymphocyte proliferation (blue arrow) and multinucleated giant cell responses were evident in the periphery.

**Figure 3:**
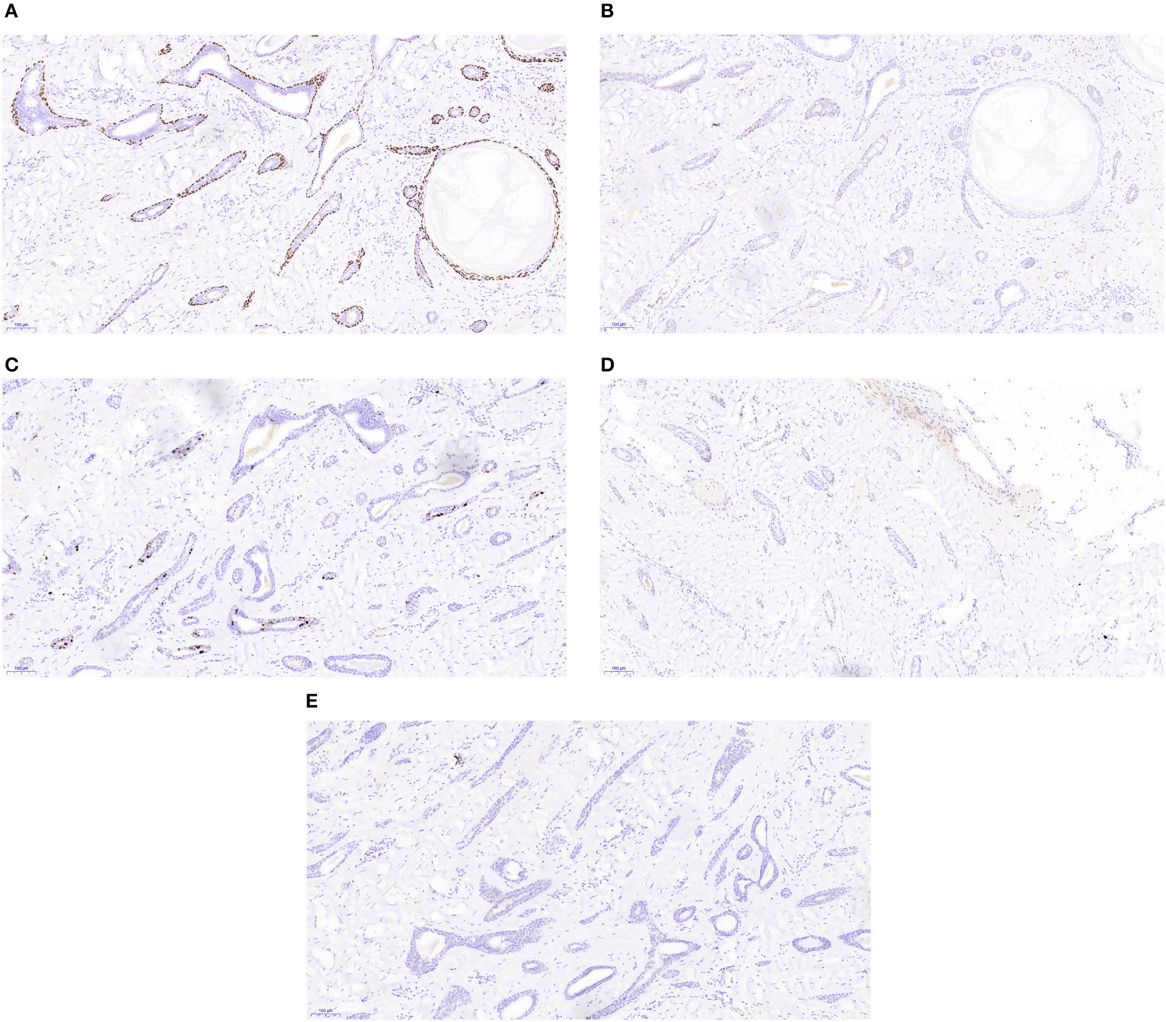
Immunohistochemistry staining of the low-grade adenosquamous carcinoma (original magnification ×100) revealed positive expression of p63 **(A)**, weakly positive expression of estrogen receptor **(B)**, low expression of Ki-67 **(C)**, and no expression of progesterone receptor **(D)** or human epidermal growth factor receptor 2 **(E)**.

Pathology analysis using ultrasound-guided core needle biopsy detected a small amount of breast tissue with duct hyperplasia, consistent with fibroid adenoma. The patient subsequently underwent right axillary mass resection under general anesthesia. Pathology analysis of intraoperatively acquired frozen sections revealed small sweat glands and sly cysts in the fibrous interstitial spaces, which was suggestive of syringomatous adenoma. This finding was inconsistent with the postoperative pathology report, which suggested LGASC (Figure 2). Pathology revealed a mass with a tan cut surface and dimensions of ~2.2 × 1.5 × 1.6 cm. Microscopically, scattered small glandular ducts and nests of squamous differentiated cells were evident in the sclerosing stroma. The glands were elongated, and exhibited an angulated comma-shaped or polliwog-shaped appearance and a disordered infiltrative pattern. The nests of squamous cells were mostly solid bands, and some of them formed keratocysts of different sizes. Mitosis was rare. Peripheral lymphocyte proliferation and multinucleated giant cell responses were evident in the periphery. The resection margins were negative. The results of quantitative immunohistochemical analysis were ER 15%, PR negative, HER2 negative, p63 positive, and Ki-67 10% (Figure 3). The patient was advised to participate in regular follow-up without further postoperative treatment.

## Discussion

LGASC is a rare type of breast cancer, and hitherto it has only been reported in females, usually those of middle age (2, 4). Physical examination typically reveals a unilateral palpable breast mass, but bilateral lesions have also been reported (6). The current report is the first of a male patient presenting with a palpable axillary mass who was ultimately diagnosed with LGASC.

Imaging of LGASC does not depict specific manifestations (5), and it is also difficult to diagnose via core needle biopsy due to the lack of typical malignant tumor cytology and the fragmentation of punctured tissue (3, 7). Pathology analysis of intraoperatively acquired frozen section can also lead to misdiagnosis due to sampling restrictions (3, 7). For these reasons a clear diagnosis of LGASC prior to surgery is rare. Postoperative pathological consequences are of high importance.

Macroscopically LGASC tumors present as firm masses with ill-defined borders and tan or pale-yellow cut surfaces (1–3). Microscopically LGASC consists of infiltrating glandular structures with varying degrees of squamous differentiation, distributed in stroma containing spindle cells. Tumor cells exhibit disorderly arrangement, and infiltrate between ducts and lobules of the mammary gland. The ducts of the glandular structure are irregularly formed, often with a comma or polliwog shape. Squamous differentiation is evident in some glands, and its characteristics include eosinophilic cytoplasm, densely arranged cells, stratification, the presence of intercellular bridges, and the formation of keratinized beads and keratinous cysts. The tumor stroma is rich in spindle cells with varying degrees of inflammatory cell infiltration (2, 5–8). In the current patient the postoperative pathology was consistent with the above-described characteristics. Immunohistochemically, myoepithelial markers are expressed to varying degrees in LGASC (9). The present patient's tumor was p63-positive. Most LGASCs are negative for ER, PR, and HER2, but Van Hoeven et al. (2) reported ER and PR expression in two cases, and Drudis et al. (10) reported HER2 expression in 46% of tumors. In the current patient the tumor cells were positive for ER, and negative for PR and HER2. This expression profile was inconsistent with the typical triple-negative immunophenotype of LGASC.

The progression of LGASC is very slow (3), and its prognosis is generally excellent with distant and lymph node metastases occurring only rarely (1, 2, 4). Notably however, no unified strategy for the treatment of LGASC has been developed. Currently it is usually managed via surgery, including local excision and mastectomy. The local recurrence rate of LGASC after local resection remains relatively high, especially in patients who undergo excisional biopsy without margin status (1, 2, 4, 5). Local resection with negative margins is therefore very important, otherwise re-excision should be performed in cases with positive margins. In cases of recurrence, re-excision can achieve satisfactory results (2). Sentinel lymph node biopsy and axillary dissection are not required, due to the rarity of lymph node metastases. To date no clinical data on the use of adjuvant therapy have been reported. Postoperative adjuvant radiotherapy can be considered when margins cannot be cleared surgically, due to the high recurrence rate after local resection, but currently the relevant evidence available in this regard is insufficient. Treatment strategies for male breast cancer are mainly based on data from small retrospective studies or they are extrapolated from the results of clinical trials for female breast cancer. Previously reported evidence derived from the treatment of LGASC, in women suggested that postoperative radiotherapy was not advisable in the current patient. Furthermore, Madden et al. (11) and Rogowski et al. (12) analyzed the benefits of postoperative radiotherapy for male breast cancer and concluded that it was of little benefit. Local resection to ensure negative margins was deemed to be of great importance in the present patient with barren breast tissue, but available evidence derived from both men and women suggested that radiotherapy after the surgery was not recommended. Therefore, the patient was advised to participate in regular outpatient follow-up without postoperative adjuvant radiotherapy.

## Conclusion

LGASC is an extremely sporadic type of breast cancer that has previously only been reported in females. The current report is the first of LGASC diagnosed in a male patient who presented with a palpable axillary mass. LGASC should be considered in male patients with mass lesions in the breast or axilla, even when core needle biopsy indicates benign lesions of breast origin. One-stage local resection is recommended for the treatment of male patients with LGASC, but it is essential to ensure that the margins are negative. Postoperative adjuvant radiotherapy is not recommended after the excision of male LGASC.

## Data Availability Statement

All datasets generated for this study are included in the article/supplementary material.

## Ethics Statement

The studies involving human participants were reviewed and approved by Ethics Committee of Taizhou Hospital of Zhejiang Province. The patients/participants provided their written informed consent to participate in this study. Written informed consent was obtained from the individual(s) for the publication of any potentially identifiable images or data included in this article.

## Author Contributions

XY and FZ acquired the data and prepared the manuscript. QW performed histological examinations with the assistance of FC. ZW and LM performed data analysis and interpretation. LC and YZ analyzed the ultrasonography images. All authors contributed to the article and approved the submitted version.

## Conflict of Interest

The authors declare that the research was conducted in the absence of any commercial or financial relationships that could be construed as a potential conflict of interest.
